# Welfare of the Medical Profession

**Published:** 1852-03

**Authors:** 


ARTICLE VIII.

### *Welfare of the Medical Profession.*

This is a part of the title of an address delivered in the Rush Medical College, by N. S. Davis, M. D., one of the Faculty of the Institution. The author is one of the originators, if not the originator, of the American Medical Association. We have a distinct recollection of his urgent appeals to the profession, and of the ability and perseverance which ultimately accomplished the great idea that he had long entertained, of concentrating the medical efforts of the United States. A man who could marshal such a force, and bring about such results, must possess original qualities; and in the discourse before us, Dr. Davis has written with energy, and a like philosophy, too, on the dignity, honor and welfare of the medical profession. He does not appear alarmed at the enormous increase of physicians; he regards, in mercantile language, the quality more than the quantity; but unless a high standard of preparation is rigidly maintained by the schools, the people may be quite as much alarmed as those who are in dread of competitors in the field of business. There are some historical memoranda introduced, which are very striking and very true, to show what has been the character of medicine in the ancient seats of civilization, and what it now is, in the dark triumphs of moslemism in those same countries. Dr. Davis could not produce an uninteresting paper, since the current of his thoughts is always indicative of activity, freshness, and a hearty determination to devote his powers to the honorable advancement of a profession, to which he is himself an honor.—*Boston Med. and Surg. Jour.*

